# Aquatic Insects and their Potential to Contribute to the Diet of the Globally Expanding Human Population

**DOI:** 10.3390/insects8030072

**Published:** 2017-07-21

**Authors:** D. Dudley Williams, Siân S. Williams

**Affiliations:** 1 Department of Biological Sciences, University of Toronto Scarborough, 1265 Military Trail, Toronto, ON M1C1A4, Canada; 2 The Wildlife Trust, The Manor House, Broad Street, Great Cambourne, Cambridge CB23 6DH, UK; sian.williams@wildlifebcn.org

**Keywords:** aquatic insects, entomophagy, human diet, animal feed, life histories, environmental requirements

## Abstract

Of the 30 extant orders of true insect, 12 are considered to be aquatic, or semiaquatic, in either some or all of their life stages. Out of these, six orders contain species engaged in entomophagy, but very few are being harvested effectively, leading to over-exploitation and local extinction. Examples of existing practices are given, ranging from the extremes of including insects (e.g., dipterans) in the dietary cores of many indigenous peoples to consumption of selected insects, by a wealthy few, as novelty food (e.g., caddisflies). The comparative nutritional worth of aquatic insects to the human diet and to domestic animal feed is examined. Questions are raised as to whether natural populations of aquatic insects can yield sufficient biomass to be of practicable and sustained use, whether some species can be brought into high-yield cultivation, and what are the requirements and limitations involved in achieving this?

## 1. Introduction

Entomophagy (from the Greek ‘entoma’, meaning ‘insects’ and ‘phagein’, meaning ‘to eat’) is a trait that we *Homo sapiens* have inherited from our early hominid ancestors. Indeed, the habit is likely to have stemmed from much earlier primate ancestors which were largely arboreal insectivores, similar to modern bushbabies and marmosets. Tangible evidence of humans eating insects lies in the discovery of insect chitin in human coproliths dating back to almost 10,000 years BP [1]. Advocacy for insects in the diet of modern man dates back to ancient Chinese classical writings more than 3000 years old [2] and, more recently in the West, to texts such as Holt (1885) [3] and Bodenheimer (1951) [4].

At present, over 2100 edible insect species have been identified [5], with over 2 billion people practicing entomophagy. This will likely swell to around 9 billion by 2050—with the most commonly consumed types comprising grasshoppers, crickets, locusts, beetles, bees, and caterpillars [6,7,8,9]. Entomophagy is most common in Africa, Columbia, Mexico, China, Thailand, Indonesia, Japan, and Australia where insects are eaten by around 3000 different ethnic groups. In the Western World, however, a perception barrier exists [10]. 

There is a considerable amount of information on the use of terrestrial insects in the human diet [7,8], but the same cannot be said for aquatic insects. The purpose of this review is to examine the known and potential nutritional worth of the latter to the human diet.

## 2. Background

The most convenient sub-grouping of insects for comparative purposes is at the level of Order. Of the 30 extant orders of true insect, 12 contain species considered to be aquatic, or semiaquatic, in either some or all of their life stages (Table 1). These orders can be divided into two groups, based on the development of individuals. The more primitive orders (Ephemeroptera to Hemiptera) show hemimetabolous development, that is, the external form of the nymph gradually approaches, through a series of instars, that of the adult; the last nymphal instar resembling the adult very closely. In the Megaloptera and all higher insect orders, the immature stages (known as larvae) do not resemble the adult so that a marked change in external appearance takes place at metamorphosis. This change, combined with an additional stage in the life cycle between the larva and adult (the pupa) denotes holometabolous development. All three stages are found in human diets. As is convention [11], we include the aquatic and semi-aquatic springtails (Hexapoda: Class Collembola) in this review.

Before we can identify potential aquatic insects for harvesting and/or cultivation, it would be prudent to examine their relevant biology and natural habitats so that their suitabilities can be assessed. Insects, in general, have become adapted to a wide range of environments, from hot to cold, from forests to grasslands, from valley bottoms to mountain tops, and from lakes, ponds and rivers to deserts. Some species feed on fresh plant material whereas others feed on decaying matter, plant or animal; others may be carnivorous or parasitic. Much of this diversity derives from the morphological and physiological adaptations that, historically, allowed their early colonization of terrestrial environments. For example, the highly chitinised exoskeleton which prevents desiccation; the small size and ability to fly, which have enhanced dispersal capabilities; the short generation time which allows rapid adaptation to shifts in environmental forces; and the ability, in the more advanced orders, to exist in two discretely functioning stages, larva and adult, which may occur in different habitats and therefore be subject to different selection processes. 

**Figure 1:**
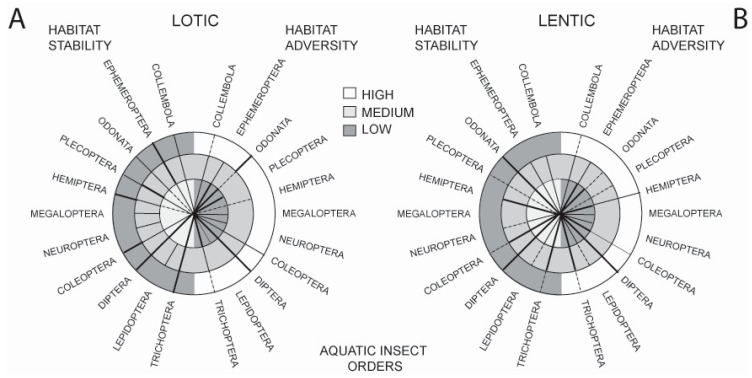
Distribution of the aquatic insect orders (including the hexapod Collembola) along two major habitat axes: *Stability* (left-hand side of the circles) and *Adversity* (right-hand side of the circles). In (**A**), orders are plotted over lotic habitats ranging from low to high stability and low to high adversity. In (**B**), orders are similarly plotted over lentic habitats. The width of the distribution lines is proportional to the importance of the order in a particular habitat type, broken lines indicate that the order is poorly represented in that habitat type (after [13]).

Such adaptations have resulted in the present-day distribution of orders across a wide spectrum of available freshwater habitats. This is illustrated in Figure 1 which shows the occurrence of the major aquatic insect orders across two strong habitat axes, stability and adversity, comparing running (lotic) and standing (lentic) waters. The stability axis shows habitats ranging from the highly stable permanent, cold freshwater springs and permanent ponds and lakes to unstable systems such as temporary streams and phytotelmata. The adversity axis shows habitats ranging from the benign (e.g., clean, cool, freshwater lotic and lentic waterbodies) to ones in which conditions for life are close to the survival limits of insects (e.g., hot springs, very high salinity, extreme pH, gross pollution and petroleum ponds). 

Observations from these comparisons are as follows: in running water habitats, mayflies, caddisflies, and dipterans are well represented in habitats of differing stability. However, caddisflies and mayflies deal less well with increasing habitat adversity. In standing water, dipterans cope well with variation in both stability and adversity, and odonates cope well with decreasing habitat stability—more so than in lotic habitats. Orders that appear not to have been able to adapt to decreasing lotic habitat stability include the Megaloptera, Neuroptera, and Lepidoptera; they are also absent from highly stable lotic habitats. Few taxa, apart from the Diptera and Odonata, are well represented in adverse lotic habitats, and this is mirrored in lentic waters where, although a number of orders can tolerate moderate adversity, only the Hemiptera and Diptera tolerate high adversity. Coleoptera live in lentic and lotic habitats that span both stability and adversity axes, but they do not occur with the frequency of some other groups.

Although, as a group, the Diptera cope well with conditions in most aquatic habitats, there are marked differences in adaptability at the Family level. In running waters, tipulids and chironomids deal particularly well with variations in habitat stability. In adverse lotic habitats, however, it is the chironomids and ephydrids that predominate. Ephydrids are abundant, too, in adverse lentic waters, and in highly- or moderately stable ones. Families that fare well across both axes are the Chironomidae, Ceratopogonidae, Chaoboridae, and Culicidae. The following brachyceran families tend to do well in heavily polluted (high adversity) standing waters rich in organic matter: Dolichopodidae (long-legged flies), Syrphidae (hoverflies), Sphaeroceridae (lesser dung flies), Scathophagidae (dung flies), Anthomyiidae (flower flies), and Sepsidae (black scavenger flies) [13]. 

Using such insect-habitat information allows identification of taxa that could be good candidates for entomophagy. For example, a species which occurs naturally in very high numbers which may be amenable to periodic harvesting from the wild, or a species that, because of high adversity tolerance, might lend itself to mass culture using organic waste as its food. Identification of insect orders that may contain candidate species are shown in Table 2. 

## 3. Details of Those Insect Orders Having Greatest Entomophagous and/or Bulk Harvesting Potential

Six insect orders are likely to contain candidate species. Their merits are as follows—note that because of the paucity of scientifically verified information, anecdotal information (from the popular press and web-based sources) is also included. Where known, nutritive values are also given. These details should be examined alongside the global list of aquatic insect species (given in the Appendix) for which there are records of entomophagy. 

A point to note is that most of the global Biological Regions support aquatic species that have been recorded as edible and that belong to the six orders. However, the Australian Region has only three orders, possibly due to less study or a more ‘Western’ attitude to eating insects in that country. The same may be true for the relatively low species count in the Nearctic.

- **Ephemeroptera**—There are more than 3000 species of mayfly. The majority are lotic, but while many species live in small to medium-sized streams, huge, natural, mass emergences have been recorded from large rivers (e.g., the Mississippi; [18]). These events are seasonal but, as the adults are attracted to lights and bridges, there are opportunities to gather them, in bulk, for processing and storage. Large lentic species, such as *Hexagenia limbata* (Ephemeridae), perhaps have the potential to be raised in culture as their development is highly temperature dependent and they feed by collecting fine-particle organic detritus. The species has been observed to complete its life cycle in 17 weeks in warm canals in Utah and, in laboratory tanks, this has been reduced to 13 weeks [19]. The possibility of rearing lotic species has been trialed, with some success, using a low-cost, ‘reversed-funnel’ method to provide water circulation [20].

There is anecdotal evidence that adult mayflies are harvested and eaten in many parts of China and Japan, and also in New Guinea and Vietnam. Both the nymphs and adults of *Ephemerella jianghongensis* are eaten in Yunnan Province, China [7]. In Malawi, people make a paste, called kungu, out of mayflies (*Caenis kungu*) mixed with mosquitoes, which is formed into dried cakes. On the shores of Lake Victoria, mayflies of the genus *Povilla* (Polymitarcyidae) are dried for subsequent use in meal preparation. This ‘insect flour’ often incorporates the bodies of chaoborid and chironomid dipterans which emerge at the same time [21]. There are also tales of 17th century Incas eating nymphs of *Euthyplocia* (Euthyplociidae) (and possibly also *Campylocia*) either raw or in a spicy sauce [22,23]. The crude protein content of dried mayfly nymphs is reported to be 66% [7].

- **Odonata**—There are almost 6000 species of odonate, distributed from the tropics, where the greatest numbers and diversity occur, to the tree-line in polar regions [13]. Nymphs of six to seven species are eaten in China, with the most common being *Crocothemis servilia*, *Gomphus cuneatus,* and *Lestes praemorsa* ([24]; see also the Appendix). In Thailand, Hanboonsong [25] recorded species from four genera as being commonly eaten (*Aeshna*, *Ceriagrion*, *Epophtalmia,* and *Rhyothemis*). In total, some 26 species of odonate are known to be eaten in the Oriental Region (see the Appendix). Of note is the preference for species of Libellulidae (17) in the Orient but for species of Aeschnidae (7) in the Neotropics (see Appendix). The crude protein content of dried dragonfly nymphs has been measured at between 40 and 65% [7].

Based on odonates alone, there is strong evidence of existing entomophagy in many parts of the Orient, and also in the Neotropics (at least 15 species eaten). In the terraced rice paddy fields of Bali, the local people catch adult dragonflies using the sticky sap from jackfruit or frangipani trees. The sap is either painted on to the tip of a thin stick or formed into a small ball which is then whirled around on a string. In both methods, individual insects are targeted and thus the yield is relatively low making them not an important food source, but offering some variety in the people’s diet. In Laos, the preferred species is *Anax guttatus* (Aeshnidae), which is captured using a candle suspended over a dish of water. Dragonfly nymphs are also eaten, but more rarely [26]. There may be some potential for suitable lentic species to be raised in tanks. However, most species have a long life cycle and the nymphs require live invertebrate food—although mass-reared mosquito larvae could provide a convenient source. 

Laboratory studies have successfully reared the lentic species *Ischnura ramburii* in large numbers (1200+) using *Drosophila melanogaster* (fruit-flies) as a food source [27]. However, the cost was estimated at $1.00 US per emerged damselfly, which could limit the potential for commercial production—although perhaps not for the specialist market.

- **Hemiptera**—Globally, there are around 3800 known species of aquatic and semiaquatic Hemiptera (grouped by some into the suborder Heteroptera) and, of these, a number are eaten. For example, there is a long history of consuming corixids in Mexico where their eggs are harvested as ‘Ahuautle’ and command high prices. Some six species (collectively known as ‘Axayacatl’) are eaten although these are now under threat due to habitat destruction [17]. The relative proportions of terrestrial and aquatic species eaten in this region are 79% and 21%, respectively.

In many parts of Asia, belostomatids (giant water bugs) are a well-liked delicacy [28], but they are also eaten in most other parts of the world [3,9]. In particular, *Lethocerus (=Kirkaldyia) indicus*, at a length of up to 12 cm, is prized in Vietnam, Thailand, and the Philippines. This species can be readily attracted to lights but is becoming rare. Researchers at Khon Kaen University in Thailand have reported some success with a new rearing technique for *L. indicus* [29]. There is also potential for some, largely lentic, hemipteran species to be raised in tanks, although production on a commercial scale has still not been achieved [30]. However, very recently there has been some success at farming *L. deyrolli* in Japan [31]. Shantibala et al. [32] have recorded consumption of *L. indicus* together with the water scorpion, *Laccotrephes maculatus* (Nepidae), amongst the indigenous peoples of Manipur, India. In Thailand, Hanboonsong [25] has recorded two species of belostomatid, the water strider *Cylindrostethus scrutator*, three species of Nepidae, and two species of backswimmer (Notonectidae: *Anisops barbutus* and *A. bouvieri*) as being commonly eaten. In Thailand, school children are encouraged to raise insects, including *L. indicus*, to promote nutrition [30]. More than half of the species consumed are collected during the rainy season (May–July) when their pond and wetland habitats are most prevalent in the landscape. Edible hemipterans particularly attracted to these temporary waterbodies include naucorids (creeping water bugs), notonectids (back-swimmers), and gerrids (water striders) [9]. During the rest of the year, villagers supplement their diets with commercial, mass-reared insects, such as crickets.

There exist several techniques for raising smaller hemipterans for research purposes in the laboratory. For example, the water strider *Limnogonus fossarum fossarum*, common in the Oriental Region, can yield more than five generations per year, provided that it is fed suitable live prey species [33]. McPherson [34] outlined a technique for rearing *Notonecta hoffmanni* in the laboratory. It is possible that some of these protocols could be scaled up for greater yield.

- **Coleoptera**—Of the close to 400,000 species of beetle, roughly 5000 (1.3%) are considered to be aquatic. The latter live in a very wide spectrum of habitats (coldwater springs to salt-marshes) but, while they may be important to these ecosystems, they do not reach the levels of density or biomass seen in other orders, such as the Trichoptera and Diptera [35]. Lotic species are unlikely candidates for mass harvesting due to typically small, dispersed natural populations (although riffle beetles, Elmidae, may be an exception; [36]). However, some lentic species can occur in quite high densities (e.g., gyrinids and dytiscids), although their abundance is often seasonal, due to life cycle characteristics and habitat availability (e.g., temporary ponds and puddles; [37]). Globally, upwards of 78 species, in 22 genera, have been recorded as being edible. Mexico leads with 36 species eaten, followed by China (26) and Japan (15) [36]. Certain genera are consumed more than others, with 22 species within the dytiscid genus *Cybister* confirmed as eaten, worldwide, and also 12 species of the hydrophilid genus *Hydrophilus* [9]. In Thailand, Hanboonsong [25] reports three species of Hydrophilidae and eight species of Dytiscidae as commonly eaten. In China, they are consumed more for their anti-diuretic effect, although *Cybister tripunctatus* has a high fat content (21.6%), which can contribute significantly as a source of oil in the diet, and also strong antioxidant properties [32]. However, this species, like a number of other exploited aquatic insects, is on the decline, and is on the Red Data List in Japan [38]. Ramos-Elorduy [17] has reported that, in Mexico, 14 insect species are considered to be threatened. In large part, this decline is due to over-harvesting of wild populations. Clearly, culturing techniques need to be developed to compensate. Such an approach has multiple benefits for local populations, not only directly through food but also for local economies, as excess beetle biomass can be sold to ready national and international markets. As a comparison, as early as 1994 in South Africa, Van der Waal showed the sale of grasshoppers to be a business worth over 1 million dollars, annually [39]. More recently in Uganda, Agea et al. [40] reported on a thriving trade based on the sale of the wild-caught grasshopper, *Ruspolia nitidula*, with the average retail price per kilogram being $2.80 U.S., comparable to the price of goat meat ($2.13). Such data are not readily available for aquatic insects. However, in Guangdong, China, water beetles sold in local markets are now being hatched in special nurseries [30,41]. Two problems hinder the mass rearing of water beetles: provision of an ample and continuous supply of live food; and surface-rippling resulting from tank aeration requirements that interfere with the respiration of small beetle larvae. Using *Dytiscus sharpi* as a model species, Inoda and Kamimura [42] have designed a new open-aquarium system that largely addresses the second issue. Adequate supply of live food can be achieved through parallel mass rearing of mosquito larvae (see next section).
- **Diptera**—There are around 120,000 known species of true (two-winged) fly, with many more thought yet to be described. Within the order are several large aquatic families that are important to natural and human-centric ecosystems: Tipulidae (craneflies); Culicidae (mosquitoes); Chironomidae (non-biting midges); and Simuliidae (blackflies). In the life cycle, the adults are typically terrestrial, with the larvae and pupae living in water [13]. In some species and habitats, population sizes can be vast and affect humans in diverse ways—for example, negatively, as vectors of disease, and, positively, as a food source.

***Tipulidae***: Craneflies occur in virtually every type of freshwater habitat and are especially abundant in shallow margins where their larvae play an important role in shredding riparian leaf litter. With over 15,000 species, the Tipulidae represents the largest family of Diptera. In temperate regions, the life cycle is typically one year which might render them unsuitable for mass rearing. However, the decent size of many species (1 to 2 cm) could make them viable especially as they have the potential to be raised in shallow, polythene-lined depressions filled with water and leaf litter. Emerging adults are easy to collect with sweep-nets and could also represent an important food source for local communities, as they do for many bird, mammal, fish, amphibian, and reptile species [43].

***Culicidae***: The 3500 or so species of culicid are typically associated with being nuisances and/or vectors of human disease. However, their global distribution, rapid development, and occurrence at very high densities make them prime candidates for entomophagy, in several ways. For example, mass emergences of adult culicids, such as those that take place on the Arctic tundra [44], but which are not yet harvested. Included, here, should also be the example of the culicid sister-group, the Chaoboridae, which are sustainably harvested on the shores of Lake Victoria, by local Luo villagers [45]. Mass rearing and harvesting of culicid larvae should be possible through scaled-up methods already proven in the laboratory (e.g., [46,47]), with the larvae being consumed either directly, or indirectly as live food for rearing other insects, such as dytiscid beetles or belostomatid bugs (e.g., [48]). 

***Chironomidae***: Non-biting midges have a global distribution and often occur at very high densities, sometimes as a consequence of human activities, for example pollution of water bodies with organic wastes. There are thought to be perhaps 20,000 species in total, with around 5000 having been formally identified and named. Chironomids are highly speciose, often exceeding 80 species at a single site, with most aquatic predators feeding on them at some stage in their lives [49]. Many species are small, with larvae less than 1 or 2 mm in length, although some may exceed 1 cm (e.g., those belonging to the haemoglobin-containing genus *Chironomus*, commonly known as ‘bloodworms’). Chironomids can be reared at high densities under laboratory conditions in 20–22 days and contain 56% crude dried protein [50]. A potential problem with mass rearing these midges is that contact with adults or larvae sometimes causes allergic reactions, particularly in farmers and fish-food handlers [51].

Chironomids are also used in a number of real-world applications including recycling of farm manure and waste-lagoon purification where, over a five month period, the larval yield was 51 kg, wet weight [52]. In Hong Kong in 1980, Shaw and Mark reported on a large (13.5 ha) chironomid farm using chicken manure as food for the larvae which were then used for fish-food, both locally and exported to North America [53]. Harvest yields averaged 25 g m^−2^ per week. Of course, entomophagy based on sewage- or manure-fed larvae may be unappetizing or unhygienic, however ‘cleansing’ through intermediate use as fish food (e.g., carp or *Tilapia*), or as a high-protein, dried supplement for pigs or chickens might be more palatable. 

Recorded instances of chironomids being eaten directly are largely anecdotal. However, Gahukar [54] cites the case where, in Africa, species of *Chironomus* have been fed to weak children in the form of ‘insect biscuits’ to gain strength. Chironomids are also eaten together with chaoborids as the adults of both emerge at the same time on the shores of Lake Victoria (see above; [21]).

***Simuliidae***: Blackflies comprise a cosmopolitan family of biting dipterans of great importance in many parts of the world as bloodsuckers and vectors of parasites such as filarial worms. The larvae and pupae are confined to running waters where they attached themselves to firm, usually smooth substrates. Different species often exhibit preferences for certain current regimes and/or substrate types, and the outlets of ponds, lakes, reservoirs, and the spillways of dams are particularly favored and productive sites [13]. There are around 1900 known species. Where conditions are ideal, for example, in shallow, fast laminar flow in lake outlet streams, larval densities can be extremely high, with Malmqvist [55] recording more than 120 larvae cm^−2^ of substrate. Collection of larvae and pupae can be done easily by brushing them off substrate surfaces into downstream nets. Despite this simple method, there appear to have been few attempts to harvest this naturally occurring biomass. Moreover, blackfly larvae readily colonize flat, artificial substrates placed in areas of fast current, providing an alternative harvesting technique. Further, adult blackflies are attracted, in large numbers, to lights at night and thus can be netted. There is a single report of blackfly larvae being eaten as a delicacy by some Karen hill-tribes in northern Thailand [56]. Because of their ability to spread parasites, blackflies should only be eaten after cooking, with the same applying to culicids.

Simuliids are known to be difficult to rear under artificial conditions. However, Raybould [57] recorded some success with *Simulium damnosum*, using a laboratory approach, and Marr [58], similarly, but using a modification to a natural breeding place.

- **Trichoptera**—Of the approximately 7000 known species of caddisfly, all but a few live in freshwater lotic or lentic habitats. Within these, they have become adapted to a wide range of conditions and, where favorable, their larvae can occur at high densities. Consequently, the adults often emerge synchronously and in large numbers, and are strongly attracted to lights. These mass emergences can be a nuisance around urban rivers and lakes (e.g., the ‘shad-fly’ emergences that occur annually, in May and June, from the St. Lawrence, Winnipeg, and Niagara rivers in Canada [59]. However, such events provide opportunities for harvesting—although, again, there are few records of this happening. A particular habitat that promotes very high larval densities of net-spinning families (e.g., the Hydropsychidae) is the fast water flowing over flat concrete surfaces at dam outflows or around hydroelectric generation stations [60]. In a manner similar to that described, above, for blackflies, concrete slabs or slates inserted into suitable rivers can replicate such habitats from which late-instar larvae and pupae can be gathered. An alternative artificial substrate is sheets of artificial turf (‘astro-turf’) to which net-spinning larvae readily attach. There is also the potential for larger, lentic species to be raised in tanks or small artificial ponds—especially those that emulate vernal woodland pools.

Despite the potential of caddisflies to be used in the human diet, by virtue of their accessibility and the size of their larvae, there are few records of entomophagy. It is practiced, however, in Japan where the larvae are boiled and then sautéed in soya sauce and sugar. This is a delicacy known as Zaza-mushi (‘zaza’ meaning ‘the sound of rushing water’, and ‘mushi’ meaning ‘insect’) [15]. The most commonly eaten species are *Stenopsyche griseipennis*, *Parastenopsyche sauteri*, and *Cheumatopsyche brevilineata* (see Appendix), and those collected from the pristine Tenryu River are particularly prized. The high production of larvae in this river is due to a high nutrient load carried down from an upstream lake [61]. There are also anecdotal records of caddisflies being eaten in Mexico and Southern Asia (Pakistan to Nepal to Sri Lanka) [62].

## 4. Aquatic Insects: Taste Versus Nutrition

It is clear from the above examples that there is a dichotomy of purpose in the consumption of aquatic insects by humans. One is largely for tasty, perhaps even ‘trendy’, snacks which supply some nutrients and are generally accompanied by aromatic spices. These treats are frequently expensive and have found their way onto the menus and ‘niche markets’ of western countries. The other purpose is as part of the staple diet of indigenous, largely poor, peoples chiefly from tropical and subtropical countries. Often the dietary contribution is seasonal, dictated by the timing of the life history stages of the insects.

As noted, compared with terrestrial insects there is relatively little information on the occurrence of aquatic insects in the human diet and the same is true for analysis of their nutritional value. To this end, Table 3 shows the average daily requirements of essential dietary components together with approximate yield potentials from eating dried terrestrial insect bodies. Several observations are noteworthy. First, the protein yield is quite high, with one cup-full (100 g) of dried insects yielding close to the daily recommended adult reference intake (ARI). Second, in contrast, the yield of carbohydrate is very low (it would require 26 cups-full, or 5.2 kg). Third, the ranges in yield potential are quite wide (especially for total fat and fiber)—presumably dependent on the type of insect analyzed. Last, roughly 1.5 to 2 cups-full would provide the total energy (in kcal) required per day. Clearly, one would not eat insects to acquire carbohydrates, but would for protein. Indeed, aquatic insects in general tend to be excellent sources of protein, for example: Ephemeroptera 66.3% of body weight; Odonata 40–65%; Hemiptera 42–73%; and Coleoptera 23–66% [32,63]. Eating insects in combination with another source of carbohydrate (such as rice, millet, or cassava) could help approach a more balanced diet. In addition to the above components, Bergeron et al. [21] and Okedi [64] found insects to be high in minerals, B-vitamins, and essential amino-acids; thus consuming insects may be beneficial. A report by the FAO found that many edible insects are good sources of minerals such as iron and zinc. Deficiencies of iron and zinc are common health disorders worldwide and insects could contribute to preventing these [8]. 

Bell et al. [69] analyzed the lipid composition of 10 freshwater invertebrate taxa (stonefly nymphs, beetle larvae, chironomid larvae, corixid and notonectid bugs, *Ecdyonurus venosus*, *Ephemerella* sp. and *Caenis* sp. (mayflies), gammarid crustaceans, and oligochaete worms) in a comparative study of natural and commercially prepared Atlantic salmon diets. They found that a dietary fatty acid composition more akin to that of the invertebrates might be beneficial for growth, development, and the prevention of pathologies in farmed parr. Consumption of such invertebrates might well be expected to bestow similar advantages to humans. In a study of the fatty acid composition of aquatic and terrestrial insects, Fontaneto et al. [70] showed that differences in the proportion of long-chain polyunsaturated fatty acids (LC-PUFA) do exist, with the latter being richer in certain omega-6 fatty acids—which are important in normal growth and development and in brain function, in humans.

Ayieko et al. [71] identified food security as a problem affecting both insect nutrition and taste. They found that in hot climates high food spoilage is commonplace. Using termites and lake-flies (dipterans and mayflies) captured along the shores of Lake Victoria, they cooked these insects in the laboratory under different conditions: baking, boiling, steam cooking under pressure. The end products (e.g., muffins, crackers, muffins, sausages, and meatloaf) yielded two positive results: firstly, the processed products were readily accepted by consumers who were earlier concerned about eating insects—as they conformed to more familiar food types; and secondly, they extended shelf-life. Moreover, the latter may benefit from certain insect properties such as antioxidant and antibacterial activity.

## 5. Aquatic Insects and Animal Feed

In humans, the available nutrients in insects have been shown to contribute to an acceptable diet. The same also applies to animal feed although, again, the data come mostly from studies on terrestrial insects [72]—however, the possible application to aquatic insects was suggested as early as 1972 [73]. Significantly, alongside grasshoppers, lake-flies can supplement essential vitamins and minerals necessary in cattle feed, including those that improve general herd and udder health (vitamins E and A, Beta-carotene, and selenium; [74]). The stark contrast between insects and cattle is most evident when their respective conversion ratios are compared—although it should be noted that cattle are not amongst the highest converters [75,76]. Cattle need up to 18 kg of feed to produce 1 kg of edible meat. Crickets require 2 kg of feed to produce 1 kg of edible meat [77]. Clearly, it would be more efficient to use insects directly as human food. 

Although use of aquatic insects is largely not yet on the feed-production radar, there is recognition that terrestrial insects can contribute. Indeed, insects have a similar market to fishmeal and are in use as feed in aquaculture and livestock, and in the pet industry. As the production costs of this feed rise (related to the decreasing supply of industrially caught fish and increase in aquaculture), the search is on for alternative and sustainable protein sources—which makes insects an attractive feed option [78]. Insects can contribute similarly to the poultry and beef industries, although there are still some financial issues as current production costs are high—Meuwissen [8] has shown that the production of mealworms is still almost 5 times as expensive as conventional chicken feed. As for aquatic species, chironomids are used as fish food and can be grown using farm wastes, such as chicken manure [53]. Given the known issues with allergic reactions (see above), there may be more potential for chironomids as animal feed than as human food.

Interestingly, farmed marine fishes require highly unsaturated fatty acids (HUFA) in their prepared diets. The latter are usually absent from terrestrial insects, but are more common in aquatic insects which feed on aquatic plants and animals that are richer in HUFA. Aquafeeds containing freshwater insects (for example, mosquitoes) would be advantageous [79].

## 6. Harvesting Versus Culturing

Based on current knowledge, it is evident that some aquatic insects are, historically, already a significant part of the diets of many indigenous peoples. Access to this edible biomass is typically via harvesting natural populations at times dictated by species availabilities. Knowledge of habitats and life cycles is fundamental for this and likely has become drawn into local folklore and tradition. However, based on this same information there exists potential for culturing edible species, using the simplest of materials and methods—such as creating artificial ponds (for attracting migrating water-beetles, or raising odonates) or placing flat tiles in running waters (for colonization by net-spinning caddisflies or blackflies). 

‘Semi-cultivation’ has been practiced historically in Mexico using the eggs of aquatic hemipterans. The method used submerged vegetation bundles set out as egg-laying substrates (akin to mussel farming) [80]. Parsons [81] estimated that an adult insect harvest of 10 kg and an egg harvest of 5 kg would have been attainable every two weeks per hectare of lake surface—a combined annual yield of almost 4000 metric tonnes, given a lake surface area of 10,000 hectares.

Unfortunately, while there are descriptions of methods of mass-rearing insects in closed environments [54], precious few involve farming aquatic species. Nevertheless, looking ahead, once techniques for obtaining aquatic insects in bulk have been developed, their yields can be subjected to the innovative processing protocols that have already been developed for terrestrial insects, such as crickets or mealworms. In 2016, the Kenya News Agency reported on a thriving facility at the Jaramogi Oginga Odinga University of Science and Technology where crickets are being successfully raised in bulk to address the problem of malnutrition in Africa. The operation plans to include other species in due course [82]. A very thorough review of this modern insect-based food industry is given by Dossey et al. [83]. Hanboonsong [25], further, points to the potential benefit of including locusts amongst the commercialized species. Not only can they be harvested in huge numbers during population outbreaks, but collecting them negates the use of pesticides. In some instances, demand has outstripped supply such that locusts are being brought into Thailand from neighboring countries, such as Cambodia [30].

## 7. Dangers in Eating Aquatic Insects

A potential negative aspect in eating, especially raw, insects is the potential for transmission of zoonotic infections. However, forms such as *Salmonella* bacteria, commonly found in poultry and beef, nematode worms (*Trichinella*), and pork, are already part of the foods that we routinely eat. Thorough heating eliminates this problem, and no significant health problems have been reported from consuming edible insects [9]. However, Rumpold and Schluter [84] caution that some insects may contain allergenic or toxic substances—the latter as a result of having eaten plants contaminated with heavy metals or other chemicals [8]. Moreover, there are suggestions that an unbalanced intake of edible insects may be associated with obesity, chronic degenerative disease, and stones in the urinary tract—possibly related to the high protein levels in insects [30].

## 8. Environmental Change

On the local scale, insect development, emergence, and swarming are known to be affected by regional weather conditions, particularly temperature, winds, and barometric pressure [85]. For example, warmer water can result in more frequent or precocial emergence, and changes in wind speed and direction can interfere with mating or emergence site location along shorelines. As the global climate warms, species populations will either adapt to these changes or become extinct. Pollution and other changes in water quality will similarly affect local and regional populations. For example, intensive agriculture leads to runoff of nutrient- and pesticide-laden water into rivers and lakes, together with suspended soil particles. On the positive side, aquatic insects may be more robust in dealing with climate change than, for example, arable crop plants [4]. Indeed, in the case of mosquitoes in the Arctic, it is predicted that a 2 °C rise in air temperature will result in a 50% increase in the already huge populations of these insects [44]—perhaps a large-scale harvesting opportunity given development of suitable collecting technology.

## 9. Conclusions

Existing and potential entomophagy in aquatic insects, together with proven and suggested harvesting methods are summarized in Table 4. Of the six orders, there are very few containing species that are being harvested effectively. Of those that are, for example beetles and hemipterans, their management is largely at a ‘hunter-gather’ stage. Sustainability has largely not yet entered the equation, and as a consequence an increasing number of species have become rare, for example the dytiscid beetle *Cybister tripunctatus* and the belostomatid *Lethocerus indicus*. Groups that currently have no or very little engagement with entomophagy are the craneflies (tipulids), biting midges (culicids), and blackflies (simuliids), but all of these have the potential to be higher, given suitable improvements in rearing technology. Groups that are currently eaten by people, albeit at a low level, are the mayflies, caddisflies, and non-biting midges (chironomids) which again, with improved methodologies, have considerably higher potential. The latter also applies to those groups with a current medium engagement with entomophagy, namely the odonates, hemipterans, beetles, and chaoborids (phantom midges). A number of these are, however, facing local extinction if harvesting methods are not broadened to include population-sustainment.

The above indicates the wide range of possibilities available for ramping up the use of aquatic insects for food. However, given the human condition and market dynamics, there will always be extremes. For example, high prices will be paid by a wealthy few for novelty foods, such as Zaza-mushi, whereas the world’s poorest may survive only by eating sewage- or manure-fed chironomid larvae whose nutrients have been transformed into a dried, high-protein feed for chickens.

To be successful, all harvesting protocols will need to have their approaches deeply rooted in a thorough understanding of the life histories and environmental requirements of individual insect species.

## Figures and Tables

**Table 1:** Higher classification of living aquatic insects (after [12]).

Class Collembola [springtails]
Class Insecta
Subclass Ptilota
Infraclass Palaeopterygota
Order Ephemeroptera [mayflies]
Order Odonata [dragonflies/damselflies]
Infraclass Neopterygota
Order Plecoptera [stoneflies]
Order Orthoptera [grasshoppers/crickets]
Order Hemiptera [true bugs]
Order Megaloptera [Dobsonflies]
Order Neuroptera [lacewings]
Order Coleoptera [beetles]
Order Diptera [true flies]
Order Lepidoptera [butterflies/moths]
Order Trichoptera [caddisflies]
Order Hymenoptera [bees/wasps/ants]

**Table 2:** Identification of aquatic insect orders that may/may not contain candidate species for entomophagy (the most likely are shown in boldface; from [13]).

Collembola—largely lentic, but small bodied; although they can occur in rafts on the water surface such populations are likely incapable of generating significant biomass. **Ephemeroptera**—see details below. **Odonata**—see details below. Plecoptera—neither stonefly adults nor nymphs are likely candidates as they grow slowly, require cool, running water and, in nature, occur in relatively small populations. However, *Pteronarcys dorsata* is a large species (5 cm in length) with a record of being eaten in the Oriental Region [9]. Orthoptera—unlikely, due to typically small, dispersed natural populations. Hydrophilic species are found in the Gryllidae, Tettigoniidae, Acrididae, Tetrigidae, and Tridactylidae. **Hemiptera**—includes terrestrial species; see details below. Megaloptera—large bodied, but in nature typically occur in small, dispersed populations; however, some anecdotal evidence of existing entomophagy involving *Protohermes grandis* in Japan [14,15]. *Acanthacorydalis orientalis* is eaten in China [16] and *Corydalus cornutus* is eaten in the neotropics [9]. Neuroptera—unlikely, due to typically small, dispersed natural populations. Only two families have aquatic species: the Sisyridae, which is wholly aquatic, and the Osmylidae in which the larvae are semi-aquatic. **Coleoptera**—see details below. **Diptera**—see details below. Lepidoptera—unlikely, due to typically small, natural populations. Most of the aquatic lepidopterans are hydrophilic and belong to the Pyralidae. Many semi-aquatic species are miners and borers in the tissues of aquatic plants. Consumption of pyralids has been recorded for the people living in the town of Tulancalco, near Mexico City [17]. **Trichoptera**—see details below. Hymenoptera—unlikely, due to relatively small populations. Several families within the suborder Apocrita contain species associated with water. The latter are small wasps and all are parasitic on various aquatic hosts, which include dipterans, beetles, bugs (especially gerrids), damselflies and caddisflies.

**Table 3:** Comparison of the average daily requirements in the human diet with potential yield from eating insects.

Dietary Component Intake (g/day)	Recommended Adult Reference	Yield Potential from Dried Insect Bodies (Range) [100 g is Roughly ½ a Cup]
Protein	50 g	20–76 g/100 g
Carbohydrate	260 g	1–5 g/100 g
Total fat	70 g	10–60 g/100 g
Fiber	30 g	12–137 mg/kg
Energy	2000 kcal/day	293–762 kcal/100 g

(Data derived from various sources, [5,6,7,8,9,10,11,12,13,14,15,16,17,18,19,20,21,22,23,24,25,26,27,28,29,30,31,32,33,34,35,36,37,38,39,40,41,42,43,44,45,46,47,48,49,50,51,52,53,54,55,56,57,58,59,60,61,62,63,64,65,66,67,68]. Note that the yield potential values are based on a variety of studies, using a range of methodologies and different insect species. As such, the yield ranges tend to be large and should be regarded as approximations only).

**Table 4:** Summary of the existing and potential entomophagic use of aquatic insects, together with possible protocols for harvesting.

Order/Family	Existing	Potential	Harvesting Protocol
Ephemeroptera	low	could be higher	netting mass emergences of adults; possible breeding of lentics in tanks and lotics in reversed-funnel systems
Odonata	medium	could be higher	individually, via sap on sticks; possible breeding of lentics in tanks
Hemiptera	med/high	could be higher	netting and attracted to lights; up-scaling of lab protocols
Coleoptera	med/high	could be higher	wild collection of adults;some captive breeding
Diptera			
Tipulidae	none	could be viable	netting adults; creation of shallow, leaf-litter-filled pools for larvae
Culicidae/	none	could be viable	netting of adults where dense
Chaoboridae	medium	viable	existing netting of adults where they occur on lake shorelines
Chironomidae	low	very high	wild collection where densities are high; up-scaling of lab-breeding protocols; waste lagoon rearing; route biomass through carp or *Tilapia* to improve aesthetics; use as animal feed for pigs, poultry, cattle
Simuliidae	very low	could be viable	collection of adults at lights; wild collecting of larvae on flat surfaces in fast water
Trichoptera	low	could be higher	wild-caught for specialist market; same as for simuliids; possibility of rearing lentic species in tanks/ponds

## Appendix

List of global aquatic insect species that have been recorded as being edible. The world regions are as follows: Tropical Africa; Australian; Neotropical; Oriental; Nearctic; Palaearctic (note that the Neotropical includes Mexico, although the northern mountains of Mexico are included in the Nearctic; China is included in the Palaearctic, although most of southern China is included in the Oriental). Records are based on those extracted from Jongema [13].

**Table:**

Order	Family	Genus	Species	Region
Coleoptera	Dytiscidae	*Cybister*	*distinctus*	Trop. Africa
		*Cybister*	*hova*	Trop. Africa
		*Cybister*	*owas*	Trop. Africa
		*Eretes*	*sticticus*	Trop. Africa
		*Rhantus*	*latus*	Trop. Africa
	Hydrophilidae	*Hydrophilus*	*senegalensis*	Trop. Africa
Diptera	Chaoboridae	*Chaoborus*	*edulis*	Trop. Africa
		*Chaoborus*	*pallidipes*	Trop. Africa
	Chironomidae	unident.		Trop. Africa
	Culicidae	unident.		Trop. Africa
Ephemeroptera	Caenidae	*Caenis*	*kungu*	Trop. Africa
	Polymitarcidae	*Povilla*	*adusta*	Trop. Africa
Hemiptera	Belostomatidae	*Belostoma*	unident.	Trop. Africa
		*Limnogeton*	*fieberi*	Trop. Africa
	Nepidae	*Nepa*	unident.	Trop. Africa
		unident.		Trop. Africa
Odonata	Libellulidae	*Trithemis*	*arteriosa*	Trop. Africa
		unident.		Trop. Africa
Coleoptera	Gyrinidae	*Aulonogyrus*	*strigosus*	Australian
Ephemeroptera	Palingeniidae	*Plethogenesia*		Australian
		unident.		Australian
Odonata	Libellulidae	unident.		Australian
	(Zygoptera)	unident.		Australian
Coleoptera	Dytiscidae	*Cybister*	*ellipticus*	Nearctic
		*Cybister*	*explanatus*	Nearctic
Diptera	Ephydridae	*Ephydra*	*cinerea*	Nearctic
		*Ephydra*	*hians*	Nearctic
		*Ephydra*	*macellaria*	Nearctic
	Rhagionidae	*Atherix*	unident.	Nearctic
	Tipulidae	*Holorusia*	*hespera*	Nearctic
		*Tipula*	*derbyi*	Nearctic
		*Tipula*	*quaylii*	Nearctic
		*Tipula*	*simplex*	Nearctic
Hemiptera	Belostomatidae	*Lethocerus*	*americanus*	Nearctic
Odonata	Aeschnidae	*Rhionaeschna*	*multicolor*	Nearctic
Plecoptera	Perlodidae	*Isoperla*	unident.	Nearctic
Coleoptera	Dytiscidae	*Cybister*	*explanatus*	Neotropical
		*Cybister*	*fimbriolatus*	Neotropical
		*Cybister*	*flavocinctus*	Neotropical
		*Cybister*	*occidentalis*	Neotropical
		*Dytiscus*	*habilis*	Neotropical
		*Dytiscus*	*marginicollis*	Neotropical
		*Laccophilus*	*apicalis*	Neotropical
		*Laccophilus*	*fasciatus*	Neotropical
		*Megadytes*	*giganteus*	Neotropical
		*Rhantus*	*atricolor*	Neotropical
		*Rhantus*	*consimilis*	Neotropical
		*Thermonectus*	*basilaris*	Neotropical
		*Thermonectus*	*marmoratus*	Neotropical
	Elmidae	*Austrelmis*	*chilensis*	Neotropical
		*Austrelmis*	*codimentarius*	Neotropical
	Gyrinidae	*Gyrinus*	*parcus*	Neotropical
		*Gyrinus*	*plicatus*	Neotropical
	Haliplidae	*Haliplus*	*punctatus*	Neotropical
		*Peltodytes*	*mexicanus*	Neotropical
		*Peltodytes*	*ovalis*	Neotropical
	Histeridae	*Hololepta*	*guidonis*	Neotropical
	Hydrophilidae	*Berosus*	uniden.	Neotropical
		*Hydrophilus*	uniden.	Neotropical
		*Tropisternus*	*mexicanus*	Neotropical
		*Tropisternus*	*sublaevis*	Neotropical
		*Tropisternus*	*tinctus*	Neotropical
Diptera	Chironomidae	unident.		Neotropical
	Culicidae	unident.		Neotropical
	Ephydridae	*Ephydra*	*hians*	Neotropical
		*Mosillus*	*tibialis*	Neotropical
	Simuliidae	*Simulium*	*rubithorax*	Neotropical
	Stratiomyidae	*Chrysoclorina*	unident.	Neotropical
	Syrphidae	*Copestylum*	*anna*	Neotropical
		*Eristalis*	unident.	Neotropical
Ephemeroptera	Baetidae	*Baetis*	unident.	Neotropical
	Ephemeridae	*Ephemera*	unident.	Neotropical
Hemiptera	Belostomatidae	*Abedus*	*dilatutus*	Neotropical
		*Abedus*	*ovatus*	Neotropical
		*Belostoma*	*micantulum*	Neotropical
		*Lethocerus*	unident.	Neotropical
	Corixidae	*Corisella*	*edulis*	Neotropical
		*Corisella*	*mercenaria*	Neotropical
		*Corisella*	*texcocana*	Neotropical
		*Graptocorixa*	*abdominalis*	Neotropical
		*Graptocorixa*	*bimaculata*	Neotropical
		*Krisousacorixa*	*Azteca*	Neotropical
		*Krisousacorixa*	*femorata*	Neotropical
	Naucoridae	*Ambrysus*	*stali*	Neotropical
		*Ambrysus*	*usingeri*	Neotropical
		*Limnocorus*	? *minutus*	Neotropical
	Notonectidae	*Notonecta*	*unifasciata*	Neotropical
Megaloptera	Corydalidae	*Corydalus*	*cornutus*	Neotropical
		*Corydalus*	unident.	Neotropical
Odonata	Aeschnidae	*Aeschna*	unident.	Neotropical
		*Anax*	unident.	Neotropical
		*Coryphaeschna*	*adnexa*	Neotropical
		*Rhionaeschna*	*brevifrons*	Neotropical
		*Rhionaeschna*	*marchali*	Neotropical
		*Rhionaeschna*	*multicolor*	Neotropical
		*Rhionaeschna*	*peralta*	Neotropical
	Coenagrionidae	*Argia*	unident.	Neotropical
	Corduliidae	*Lauramacromia*	*dubitalis*	Neotropical
	Gomphidae	*Agriogomphus*	unident.	Neotropical
		*Progomphus*	unident.	Neotropical
		*Zonophora*	unident.	Neotropical
	Libellulidae	*Brechmorhoga*	unident.	Neotropical
		*Dasythemis*	unident.	Neotropical
	Megapodagrionidae	*Oxystigma*	unident.	Neotropical
Trichoptera	Calamoceratidae	*Phylloicus*	unident.	Neotropical
	Hydropsychidae	*Leptonema*	unident.	Neotropical
	Leptoceridae	*Oecetis*	*disjuncta*	Neotropical
		*Triplectides*	unident.	Neotropical
	Odontoceridae	*Marilia*	unident.	Neotropical
Coleoptera	Dytiscidae	*Copelatus*	unident.	Oriental
		*Cybister*	*guerini*	Oriental
		*Cybister*	*limbatus*	Oriental
		*Cybister*	*rugosus*	Oriental
		*Cybister*	*tripunctatus*	Oriental
		*Dytiscus*	unident.	Oriental
		*Eretes*	*sticticus*	Oriental
		*Hydaticus*	*rhantoides*	Oriental
		*Laccophilus*	*pulicarius*	Oriental
		*Rhantaticus*	*congestus*	Oriental
	Haliplidae	unident.		Oriental
	Hydrophilidae	*Hydrobiomorpha*	*spinicollis*	Oriental
		*Hydrophilus*	*acuminatus*	Oriental
		*Hydrophilus*	*bilineatus*	Oriental
		*Hydrophilus*	*cavisternum*	Oriental
		*Hydrophilus*	*hastatus*	Oriental
		*Hydrophilus*	*olivaceus*	Oriental
		*Hydrophilus*	*picicornis*	Oriental
		*Hydrophilus*	*pallidipalpis*	Oriental
Ephemeroptera	Baetidae	*Cloeon*	*kimminsi*	Oriental
	Ephemeridae	*Ephemera*	unident.	Oriental
Hemiptera	Belostomatidae	*Diplonychus*	unident.	Oriental
		*Lethocerus*	*indicus*	Oriental
		*Sphaerodema*	*molestum*	Oriental
		*Sphaerodema*	*rusticum*	Oriental
	Gerridae	*Cylindrostethus*	*scrutator*	Oriental
		*Gerris*	*spinole*	Oriental
	Nepidae	*Laccotrephes*	*griseus*	Oriental
		*Laccotrephes*	*maculatus*	Oriental
		*Laccotrephes*	*robustus*	Oriental
		*Laccotrephes*	*ruber*	Oriental
		*Nepa*	unident.	Oriental
		*Ranatra*	*longipes thai*	Oriental
		*Ranatra*	*varipes*	Oriental
	Notonectidae	*Anisops*	*barbata*	Oriental
		*Anisops*	*bouvieri*	Oriental
		*Notonecta*	unident.	Oriental
Odonata	Aeschnidae	*Aeschna*	unident.	Oriental
		*Anax*	*guttatus*	Oriental
	Coenagrionidae	*Ceriagrion*	unident.	Oriental
		*Enallagma*	unident.	Oriental
	Corduliidae	*Epophthalmia*	*vittigera*	Oriental
	Gomphidae	*Ictinogomphus*	*rapax*	Oriental
		? *Stylurus*	unident.	Oriental
		unident.		Oriental
	Libellulidae	*Acisoma*	*panorpoides*	Oriental
		*Brachythemis*	*contaminate*	Oriental
		*Cratilla*	*lineata*	Oriental
		*Crocothemis*	*servillia*	Oriental
		*Diplacodes*	*trivialis*	Oriental
		*Libellula*	*puchella*	Oriental
		*Neurothemis*	*ramburii*	Oriental
		*Orthetrum*	*glaucum*	Oriental
		*Orthetrum*	*sabina*	Oriental
		? *Pachydiplax*		Oriental
		*Pantala*	*flavicens*	Oriental
		*Potamarcha*	*obscura*	Oriental
		*Rhyothemis*	unident.	Oriental
		*Sympetrum*	unident.	Oriental
		*Tramea*	*transmarina*	Oriental
		*Trithemis*	*aurora*	Oriental
		? *Urothemis*		Oriental
	Macromiidae	*Macroma*	unident.	Oriental
Plecoptera	Pteronarcyidae	*Pteronarcys*	*dorsata*	Oriental
	Nemouridae	*Nemoura*	unident.	Oriental
Coleoptera	Dytiscidae	*Agabus*	*fulvipennis*	Palaearctic
		*Cybister*	*bengalensis*	Palaearctic
		*Cybister*	*brevis*	Palaearctic
		*Cybister*	*guerini*	Palaearctic
		*Cybister*	*japonicas*	Palaearctic
		*Cybister*	*lewisianus*	Palaearctic
		*Cybister*	*limbatus*	Palaearctic
		*Cybister*	*sugillatus*	Palaearctic
		*Cybister*	*tripunctatus*	Palaearctic
		*Dytiscus*	*habilis*	Palaearctic
		*Dytiscus*	*marginalis*	Palaearctic
		*Dytiscus*	*validus*	Palaearctic
		*Platambus*	*guttulus*	Palaearctic
		*Rhantus*	*pulverosus*	Palaearctic
	Gyrinidae	*Gyrinus*	*curtus*	Palaearctic
		*Gyrinus*	*japonicas*	Palaearctic
		*Dineutes*	*marginatus*	Palaearctic
	Hydrophilidae	*Hydrophilus*	*acuminatus*	Palaearctic
		*Hydrophilus*	*bilineatus*	Palaearctic
		*Hydrophilus*	*cavisternum*	Palaearctic
		*Hydrophilus*	*hastatus*	Palaearctic
		*Hydrophilus*	*pallidipalpes*	Palaearctic
		*Tropisternus*	*collaris*	Palaearctic
Diptera	Tipulidae	*Tipula*	*paludosa*	Palaearctic
Ephemeroptera	Baetidae	*Cloeon*	*dipterum*	Palaearctic
	Ephemerellidae	*Ephemerella*	*jinghongensis*	Palaearctic
Hemiptera	Belostomatidae	*Lethocerus*	*deyrollei*	Palaearctic
		*Lethocerus*	*indicus*	Palaearctic
		*Sphaerodema*	*rusticum*	Palaearctic
	Nepidae	*Laccotrephes*	*japonensis*	Palaearctic
		*Ranatra*	*chinensis*	Palaearctic
		*Ranatra*	*unicolor*	Palaearctic
Megaloptera	Corydalidae	*Acanthacorydalis*	*orientalis*	Palaearctic
		*Protohermes*	*grandis*	Palaearctic
Odonata	Gomphidae	*Gomphus*	*cuneatus*	Palaearctic
	Lestidae	*Lestes*	*praemorsus*	Palaearctic
	Libellulidae	*Crocothemis*	*servilia*	Palaearctic
		*Sympetrum*	*darwinianum*	Palaearctic
		*Sympetrum*	*eroticum*	Palaearctic
		*Sympetrum*	*infuscatum*	Palaearctic
Plecoptera	Perlidae	*Kamimuria*	*tibialis*	Palaearctic
		*Paragnetina*	*tinctpennis*	Palaearctic
Trichoptera	Hydropsychidae	*Cheumatopsyche*	*brevilineata*	Palaearctic
	Stenopsychidae	*Parastenopsyche*	*sauteri*	Palaearctic
		*Stenopsyche*	*griseipennis*	Palaearctic
